# 12/15-lipoxygenase orchestrates the clearance of apoptotic cells and maintains immunologic tolerance

**DOI:** 10.1186/1479-5876-9-S2-P5

**Published:** 2011-11-23

**Authors:** Stefan Uderhardt, Martin Herrmann, Olga Oskolkova, Reinhard Voll, Falk Nimmerjahn, Valery Bochkov, Georg Schett, Gerhard Kroenke

**Affiliations:** 1 Internal Medicine 3, University of Erlangen, Germany; 2 Vascular Biology, Medical University of Vienna, Vienna, Austria; 3 Dept. of Genetics, University of Erlangen, Germany

## 

The coordinated and non-inflammatory phagocytosis of apoptotic cells is crucial to maintain immunological tolerance. During inflammation, however, ingestion of apoptotic material by inflammatory phagocytes can provoke a break in self-tolerance. Hence, and though poorly understood, mechanisms governing the sorting of apoptotic cells into distinct and differentially-activated subsets of phagocytes are essential to prevent autoimmunity.

Here we identify the enzyme 12/15-lipoxygenase (12/15-LO) as a crucial factor orchestrating the clearance of apoptotic cells under inflammatory conditions. During peritonitis, the ingestion of apoptotic cells is confined to a distinct population of alternatively-activated, 12/15-LO-expressing resident macrophages (resMΦ). Deletion of 12/15-LO changed this pattern and neighbouring inflammatory macrophages (infMΦ) start to engulf apoptotic cells.

We hypothesized that resMΦ exert a paracrine and 12/15-LO-mediated inhibitory activity on the phagocytic capacity of infMΦ and identified oxidation products of phosphatidyl ethanolamine (oxPE) as the corresponding mediators. oxPE is generated by the action of 12/15-LO in resMΦ and consequently exposed on the macrophages' surfaces. Here, oxPE binds, blocks and scavenges soluble MFG-E8 protein and thereby selectively blocks the major pathway involved in the uptake of apoptotic cells into infMΦ. In turn, we observed a break in self-tolerance and lupus-like autoimmune disease in aged 12/15-LO-deficient mice. These mice displayed spontaneous production of autoantibodies and glomerulonephritis, which both exacerbated after apoptotic challenge in the pristane-induced model of experimental murine lupus.

Our data point towards a so far unrecognized role for enzymatic lipid oxidation during the maintenance of self-tolerance and identify a mechanism, which orchestrates the cell- and context-specific uptake of antigens by different subsets of phagocytes, imposing a new paradigm in our understanding of the clearance of apoptotic cells.

